# Muscle satellite cell heterogeneity and self-renewal

**DOI:** 10.3389/fcell.2014.00001

**Published:** 2014-01-30

**Authors:** Norio Motohashi, Atsushi Asakura

**Affiliations:** Department of Neurology, Paul and Sheila Wellstone Muscular Dystrophy Center, Stem Cell Institute, University of Minnesota Medical SchoolMinneapolis, MN, USA

**Keywords:** skeletal muscle, myogenesis, satellite cells, self-renewal, muscle regeneration, MyoD, Pax7, Myf5

## Abstract

Adult skeletal muscle possesses extraordinary regeneration capacities. After muscle injury or exercise, large numbers of newly formed muscle fibers are generated within a week as a result of expansion and differentiation of a self-renewing pool of muscle stem cells termed muscle satellite cells. Normally, satellite cells are mitotically quiescent and reside beneath the basal lamina of muscle fibers. Upon regeneration, satellite cells are activated, and give rise to daughter myogenic precursor cells. After several rounds of proliferation, these myogenic precursor cells contribute to the formation of new muscle fibers. During cell division, a minor population of myogenic precursor cells returns to quiescent satellite cells as a self-renewal process. Currently, accumulating evidence has revealed the essential roles of satellite cells in muscle regeneration and the regulatory mechanisms, while it still remains to be elucidated how satellite cell self-renewal is molecularly regulated and how satellite cells are important in aging and diseased muscle. The number of satellite cells is decreased due to the changing niche during ageing, resulting in attenuation of muscle regeneration capacity. Additionally, in Duchenne muscular dystrophy (DMD) patients, the loss of satellite cell regenerative capacity and decreased satellite cell number due to continuous needs for satellite cells lead to progressive muscle weakness with chronic degeneration. Thus, it is necessary to replenish muscle satellite cells continuously. This review outlines recent findings regarding satellite cell heterogeneity, asymmetric division and molecular mechanisms in satellite cell self-renewal which is crucial for maintenance of satellite cells as a muscle stem cell pool throughout life. In addition, we discuss roles in the stem cell niche for satellite cell maintenance, as well as related cell therapies for approaching treatment of DMD.

## Introduction

Skeletal muscle is the most abundant tissue in the mammalian body accounting for approximately 40% of body weight, and is composed of multinucleated fibers that contract to generate force and movement. In addition, skeletal muscle possesses a remarkable ability to regenerate, and can go through rapid repair following severe damage caused by exercise, toxins or diseases. Muscle satellite cells were discovered by Alexander Mauro in 1961 (Mauro, 1961), and the research about satellite cells has been stimulated to clarify the regulatory mechanisms of muscle regeneration. This has contributed to the development of therapeutic approaches for several muscular diseases including Duchenne muscular dystrophy (DMD).

Satellite cells comprise 30–35% of all muscle fiber nuclei in postnatal mouse muscle, whereas the number decreases to 2.5–6% in adult muscles (Schultz, 1974; Hawke and Garry, 2001). Based on electron microscopic analysis, satellite cells are located beneath the basal lamina and adjacent to the plasma membrane of muscle fibers, and are in a mitotically quiescent state (Mauro, 1961). Upon muscle injury, satellite cells are activated, driven out of their quiescent states, and start to proliferate. Proliferating satellite cells, termed myogenic precursor cells or myoblasts, then stop their proliferation, undergo differentiation into myocytes, and fuse either with each other or existing myofibers in order to repair injured muscle (Charge and Rudnicki, 2004). While forming myotubes, a minor fraction of satellite cells generate themselves or self-renew, and eventually return to a quiescent state as satellite cells under normal physical conditions (Collins et al., 2005). This capacity, that is the ability to maintain the number of satellite cells ready to participate in repetitive muscle regeneration, is an essential characteristic of these cells. Discovery of satellite cells, together with recent advances in techniques of molecular biology, cell biology, and genetics, have significantly contributed to clarification of the molecular and cellular mechanisms of skeletal muscle regeneration. Here we present an overview of current knowledge of muscle regeneration with a focus on satellite cells, along with a summary of the molecular processes governing satellite cell self-renewal.

## Muscle satellite cells

Satellite cells are characterized by the expression of the paired type homeobox transcription factor, Pax7, which was identified as the first quantifiable marker for satellite cells in both the quiescent and activated states and essential for satellite cell development and survival (Seale et al., 2000; Oustanina et al., 2004; Kuang et al., 2006; Lepper et al., 2009). Pax7 and the closely related Pax3, which is also expressed in quiescent satellite cells in some muscles including diaphragm (Montarras et al., 2005; Relaix et al., 2006; Hirai et al., 2010), play key roles in maintaining the proliferation of progenitors and preventing early myogenic differentiation and apoptotic cell death. The myogenic basic helix-loop-helix (bHLH) transcription factor, myogenic factors consisting of MyoD, Myf5, MRF4 and myogenin, play essential roles in myogenic specification, differentiation, and maintenance during muscle development and regeneration (Tapscott, 2005). MyoD specifically serves as a potent myogenic master transcription factor that can reprogram many non-muscle cell types to a myogenic lineage when expressed in those cells (Weintraub et al., 1989). In adult skeletal muscle, Myf5 is detected in the majority of quiescent satellite cells (Cornelison and Wold, 1997). However, Myf5 mRNA, together with microRNA-31 which suppresses Myf5 translation, is sequestered in mRNP granules present in the quiescent satellite cells, and thus protein translation of Myf5 is suspended (Crist et al., 2012). In quiescent satellite cells, both MyoD mRNA and protein are not detected (Cornelison et al., 2000). Upon muscle injury, satellite cells initiate the myogenic program by the expression of MyoD, withdraw from their quiescent state, and enter into the cell cycle as activated satellite cells. These proliferating activated satellite cells are termed myogenic precursor cells or myoblasts. During satellite cell activation, mRNP granules are dissociated, relative levels of miR-31 are reduced, and Myf5 protein accumulates, which causes the initiation of the myogenic program in satellite cells (Crist et al., 2012). Additionally, during muscle regeneration, satellite cells can be distinguished based on Pax7 and MyoD protein expression. Pax7(+)MyoD(−) cells are in a quiescent state, Pax7(+)MyoD(+) cells are in a proliferating state, and Pax7(−)MyoD(+) cells are undergoing myogenic differentiation followed by cell fusion to generated multinucleated myofibers (Olguin and Olwin, 2004; Zammit et al., 2004). In addition to cell proliferation and myofiber differentiation, a minor fraction of activated satellite cells give rise to Pax7(+)MyoD(−) non-dividing mononucleated cells, which revert to a quiescent state as a quiescent satellite cell (referred to as reserve cells for *in vitro*) (Baroffio et al., 1996; Yoshida et al., 1998). This is an important function in maintaining their own satellite cell reserve pool through self-renewal, which is essential for continuous muscle regeneration throughout the life of an animal. Collins et al. have first identified that satellite cells can generate a large number of satellite cells and myonuclei after transplantation of single myofibers into mouse regenerating muscle, and that satellite cells are self-sufficient as a source of myogenic cells (Collins et al., 2005). These observations clearly demonstrated that satellite cells function as true muscle stem cells.

## Satellite cell heterogeneity

**Figure 1:**
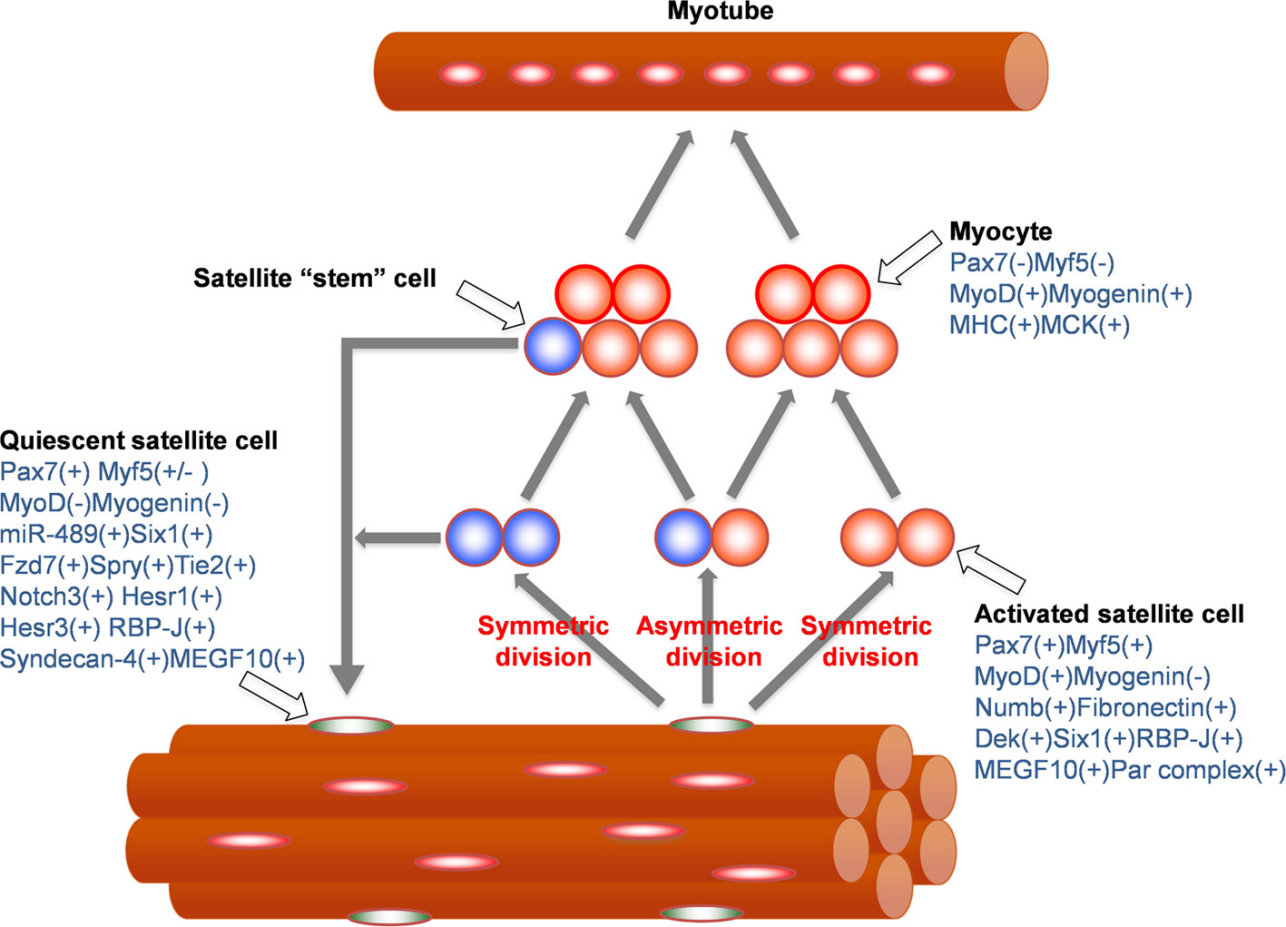
**Molecular markers for quiescent satellite cells, activated satellite cells, and myocytes**. Quiescent satellite cells are activated by signals from muscle injury and start cell division which include symmetric and asymmetric divisions to produce activated satellite cells and self-renewing satellite cell-stem cells. After several round of cell division, activated satellite cells (myogenic precursor cells or myoblasts) exit their cell cycles and give rise to myocytes which fuse each other to form multinucleated myotubes. Markers expressed in each cell types are summarized (blue letters).

Since most satellite cells are expressing Pax7, these cells were likely considered to be a homogeneous population as muscle progenitor cells. However, the accumulating evidence through gene expression profiling and cell surface marker analysis has revealed that satellite cells are a heterogeneous population (Figure 1). In addition to Pax7, large numbers of satellite cells also express Myf5 (Cornelison and Wold, 1997), M-cadherin (Irintchev et al., 1994), α7-integrin (Gnocchi et al., 2009), CD34 (Beauchamp et al., 2000), Syndecan-3/4 (Cornelison et al., 2001) or calcitonin receptor (Fukada et al., 2007). According to a recent report from Beaucamp et al. a subpopulation of satellite cells do not express Myf5, CD34 or M-cadherin (Beauchamp et al., 2000). Additionally, recent studies using *Myf5-nLacZ* mice, in which the *nLacZ* gene is inserted into the *Myf5* locus, and thus expression of nLacZ recapitulates endogenous *Myf5* mRNA expression, revealed that approximately 10% of quiescent satellite cells are LacZ(−), indicating the heterogeneity of quiescent satellite cells (Kuang et al., 2007). To support this, RT-PCR based gene expression studies in single satellite cells demonstrated that a part of Pax7(+) satellite cells express Pax3 and/or MyoD (Sacco et al., 2008).

The characteristics of satellite cells are also distinguished depending on muscle types with the distinct differences of gene expression and cell behavior *in vitro* and *in vivo*: gene expression profiling experiments have revealed the different characteristics between satellite cells from head muscles and other muscles (Harel et al., 2009), or extraocular muscle and pharyngeal muscle (Sambasivan et al., 2009). Ono et al. have also reported the gene expression differences as well as the distinct stem cell abilities in satellite cells from extensor digitorum longus (EDL) and masseter muscle (Ono et al., 2010). These observations clearly indicated that satellite cells from different muscle types are not only genetically but also functionally heterogeneous populations, and that their different characteristics of satellite cells from different muscle types might be governed by their developmental origins, environments, and niches.

In order to evaluate functional heterogeneity of satellite cells, several recent studies have demonstrated multiple transplantation experiments. Collins et al. elegantly performed an experiment wherein single myofibers with satellite cells were isolated from mouse muscle, and then transplanted into radiation-ablated muscles of *mdx*/*nude* mice (Collins et al., 2005). A single myofiber with a few satellite cells gave rise to a large number of myofibers as well as self-renewed satellite cells. In addition, the number of myofibers generated by tibialis anterior (TA) muscle was significantly less than those formed from EDL or soleus (SOL) muscle (Collins et al., 2005). These observations strongly suggest that satellite cells possess intrinsically different properties depending on the origin of muscle. Sacco et al. conducted the experiments that quiescent satellite cells [CD45(−) CD11b(−) CD31(−) Sca1(−) α7-integrin(+) CD34(+) cells] were isolated from adult muscle by FACS, and single quiescent satellite cells were transplanted into irradiated muscle. As a result, they found that a single satellite cell has a remarkable ability of proliferation and differentiation, and further revealed that some transplanted satellite cells generated Pax7-expressing satellite cells after engraftment (Sacco et al., 2008). These findings are strong evidence that satellite cell populations are heterogeneous and exhibit high potency of self-renewal *in vivo*.

Increasing evidence has revealed the exhibition of “stemness” potential in satellite cells described above, while several studies have proposed that satellite cells are composed of two different populations: one population consists of myogenic satellite cells, which possess myogenic differentiation potential. The other population is an undifferentiated subgroup, which retains the satellite stem cell profile (Collins et al., 2005; Kuang et al., 2007; Ono et al., 2010). Previous studies proposed that a small subset of satellite cells maintain an undifferentiated subpopulation and remain in a quiescent state during myogenic progression (Yoshida et al., 1998). Additionally, the majority of satellite cells are a highly proliferative, whereas the remaining number of satellite cells divide slowly (Schultz, 1996; Shinin et al., 2006). These observations suggest that the slow-dividing minor population exhibits the high potency to self-renew whereas the fast-proliferating cell population tends to undergo myogenic differentiation. This hypothesis was supported by a recent study in which satellite cells could be distinguished as fast and slow-dividing cells through labeling with fluorescent lipophilic dye (PKH26) (Ono et al., 2012). Fast dividing cells gave rise to a higher number of myogenic differentiated cells while slow dividing cells represented quiescent-like self-renewing cells (Ono et al., 2012). In addition, transplantation experiments demonstrate that slow dividing cells possess stem cell like potential, and contribute to continuous muscle regeneration *in vivo*, suggesting that slow dividing myogenic cells are able to produce myogenic stem cell progeny (Ono et al., 2012). In this study, gene expression analysis in slow dividing cells demonstrated some important clues in distinguishing the “stem cell” population from the myogenic population. As a next step, the identification and isolation of slow-dividing “stem cell” like populations will be required.

Muscle satellite cells mainly contribute to muscle regeneration while several studies have revealed that non-satellite cells, such as bone marrow derived stem cells (Dreyfus et al., 2004; Dezawa et al., 2005), side population (SP) cells (Gussoni et al., 1999; Asakura et al., 2002), mesoangioblasts/pericytes (Dellavalle et al., 2007) and CD133+ cells (Torrente et al., 2004), are able to contribute in myotube formation as well as satellite cell reconstitution during muscle regeneration. These cells do not initially express Pax7. However, once they enter in the satellite cell niche, they can fuse with other myogenic cells to form muscle fibers. In addition, these cells are also able to differentiate into Pax7-expressing cells, indicating that non-satellite cells also form muscle satellite cells. These phenomena also may attribute to muscle satellite cell heterogeneity.

## Satellite cell self-renewal and the molecular mechanisms

Satellite cell differentiation is essential to provide newly formed myofibers while satellite cell self-renewal is also essential to replenish the satellite cell pool. Maintenance of this balance between satellite cell differentiation and self-renewal is required for muscle homeostasis. A defect in self-renewal ability leads to a decrease in satellite cell number, resulting in depletion of the satellite cell pool as well as in reduced muscle regeneration capacity. The mechanisms regulating satellite cell self-renewal are exhibited in two different manners of cell division; one is by asymmetric division, in which a major population of cells generates daughter cells committed to myogenic differentiation whereas small population cells give rise to self-renewing daughter cells. The other is through symmetric division, in which one stem cell population generates two identical functional “stem” daughter cells. Current studies have carefully demonstrated the self-renewal processes for satellite cells by asymmetric and/or symmetric cell division, and proposed the mechanism of self-renewal that is governed by gene expression, niche, and microenvironment.

It is well described that the myogenic determination factors Myf5, MyoD, MRF4, and Myogenin, play essential roles in myogenic specification, differentiation and maintenance during muscle development and regeneration, and their expression largely contributes to the determination of myogenic cell fate (Weintraub et al., 1991). In addition, Pax3 and Pax7 also play essential roles in myogenic specification, differentiation, and maintenance during muscle development and regeneration as an upstream transcription factor of myogenic determination factors (Lagha et al., 2008). Genetic ablation experiments demonstrated that *Pax7* gene knockout (KO) mice display reduced significant reduction in satellite cell number, resulting in the failure of muscle growth and neonatal lethality of most *Pax7* KO mice (Seale et al., 2000; Oustanina et al., 2004; Kuang et al., 2006). Following a cardiotoxin-induced skeletal muscle injury, the *Pax7* KO mutant displayed significantly reduced muscle regeneration capacity. These results strongly indicate that *Pax7* is essential for normal skeletal muscle growth and regeneration through the maintenance and regulation of muscle satellite cells (Oustanina et al., 2004; Kuang et al., 2006). Spontaneous *Pax3*-mutant (*Splotch*) mice display neural tube and cardiac chamber malformations, missing limb muscles which originate from migratory myogenic cells from somites, and die embryonically by day E14 (Borycki et al., 1999). In order to elucidate the functional role of Pax3 and Pax7 during muscle regeneration, inducible transgenic mouse systems to conditionally remove Pax3 and Pax7 in development and muscle regeneration have been generated (Lepper et al., 2009). Interestingly, *Pax3/Pax7* conditional double-mutant mice have demonstrated that Pax7 is only essential for satellite cell maintenance in juvenile mice, while adult satellite cells do not require either *Pax3* or *Pax7* for muscle regeneration (Lepper et al., 2009). However, a more recent publications from several groups demonstrated that continuous inactivation of *Pax7* induces cell cycle arrest, myogenic differentiation, and impairment of muscle regeneration *in vivo*, concluding that Pax7 is an essential factor for satellite cell maintenance in adult muscle (Murphy et al., 2011; Sambasivan et al., 2011; von Maltzahn et al., 2013).

Current studies have elucidated which genes essentially regulate muscle satellite cell self-renewal. Interestingly, heterozygous *Myf5* gene KO (*Myf5*^+/−^) satellite cells demonstrated the equivalent regenerative potency compared to wild-type satellite cells (Gayraud-Morel et al., 2012). However, *Myf5*^+/−^satellite cells exhibited high potency of self-renewal capacity compared to wild-type cells, while Pax7 heterozygous mutant satellite cells did not show any differences for self-renewal capacity compared to wild-type cells (Gayraud-Morel et al., 2012). Taken together, these data suggest that Myf5 is one of the key modulators of satellite cell self-renewal (Gayraud-Morel et al., 2012). As previously described, quiescent satellite cells [Pax7(+) MyoD(−)] rapidly initiate to express MyoD soon after satellite cell activation *in vitro* (Zammit et al., 2004). Most Pax7(+)MyoD(+) activated satellite cells or myoblasts undergo Pax7(−)MyoD(+)Myogenin(+) myocyte differentiation, whereas a subset of Pax7(+)MyoD(+) myoblasts down-regulate MyoD expression and return into Pax7(+)MyoD(−) reserve cells, which are in a quiescent state and are considered to be an equivalent cell population to quiescent satellite cells (Yoshida et al., 1998; Zammit et al., 2004). These cells have the potential to re-enter the cell cycle under growth conditions and can eventually give rise to differentiating myocytes and self-renewing reserve cells (Kitzmann et al., 1998). Therefore, the subset's reserve cells and quiescent satellite cells are currently considered satellite stem cells, and down-regulation of MyoD is essential for the self-renewal process. However, it has not been fully elucidated how reserve cells are generated from myoblast cultures. Recently, we have reported that engraftment and survival efficiency of myoblasts isolated from adult mice lacking the MyoD gene (*MyoD*^−/−^) was significantly increased compared to wild-type myoblasts following intramuscular injection into regenerating mouse muscle (Asakura et al., 2007). In addition, *MyoD*^−/−^ muscle contains an increased number of satellite cells (Megeney et al., 1996; Cornelison et al., 2000; Hirai et al., 2010), suggesting that transplanted *MyoD*^−/−^ myoblasts, but not wild-type myoblasts, could give rise to the satellite cell compartment in muscle. Importantly, the *MyoD*^−/−^ myoblasts were much more resistant to apoptosis compared to wild-type myoblasts (Hirai et al., 2010). Therefore, *MyoD*^−/−^ wild-type myoblasts preserve stem cell characteristics including resistance to apoptosis, efficient engraftment and contribution to the satellite cell compartment following transplantation. Taken together, these observations strongly suggested that down-regulation of MyoD expression is a key event for the satellite cell self-renewal process, and identification of genes and factors that repress MyoD expression may be important for the clarification of satellite cell pool maintenance mechanisms. Importantly, this anti-apoptotic property of *MyoD*^−/−^ myoblasts is mediated by up-regulation of Pax3. Pax3 works as an anti-apoptotic factor in myoblasts in stress-induced environments and during satellite cell activation and myogenic differentiation by transcriptional activation of anti-apoptotic genes, Bcl-2 and Bcl-xl. During satellite cell activation, expression of MyoD and MyoD-induced microRNA-1 (miR-1) and -206 (miR-206) are up-regulated, while Pax3 is down-regulated by miR-1 and miR-206 via direct binding of these miRNAs to the 3' untranslated region (UTR) of Pax3. This Pax3 down-regulation in activated satellite cells is also mediated by miR-27b (Crist et al., 2009). Interestingly, Pax3 transcripts are subject to alternative polyadenylation, resulting in multiple transcripts. One transcript contains a shorter 3'UTR that has a miR-27b binding site but lacks miR-1 and miR-206 binding sites. Another transcript contains a longer 3'UTR that contains miR-1 and miR-206 binding sites but lacks miR-27b binding site (Crist et al., 2009). These data may reflect a general role of alternative polyadenylation in miRNA-mediated gene regulation in stem cell function.

Recent evidence has revealed that satellite cells initiate to self-renew after an initial cell division during muscle regeneration, and that the mechanism of asymmetric cell division is regulated by the p38 α/β MAP Kinase (MAPK) pathway (Troy et al., 2012). This pathway is, as previously reported, activated in response to muscle injury to induce MyoD expression, resulting in the activation of satellite cells that then undergo myogenic differentiation (Jones et al., 2005). During myoblast proliferation, one daughter cell asymmetrically expressing Par complex, including Par-3 and Protein Kinase-Cλ (PKCλ), activated the p38 α/β MAPK signaling leading to MyoD expression, and promoted myoblast proliferation. While other daughter cells absent for Par complex did not express MyoD and generated self-renewed quiescent satellite cells (Jones et al., 2005). Moreover, it was revealed that PKCλ is required for MyoD-dependent myogenic progression, and that Par-3 knockdown (KD) promoted the generation of satellite cell self-renewal, suggesting that regulation of p38 α/β MAPK mediated by Par complex is deeply-involved in asymmetric cell division and choice between self-renewal and myogenic differentiation (Jones et al., 2005).

Additionally, recent studies have presented the several mechanisms of myoblast self-renewal. MiR-489, which is highly expressed in quiescent satellite cells and decreased in activated satellite cells, functions as the regulator of the satellite cell fate that leads back to the quiescent state by suppressing the expression of the oncogene Dek (Cheung et al., 2012). In quiescent satellite cells, miR-489 is high but Dek is absent. By contrast, down-regulation of miR-489 induces the activation of satellite cells through the up-regulation of Dek expression. These data suggest that the miR-489-Dek pathway is important for maintenance of quiescent satellite cells. Sprouty-1 (Spry1), a receptor tyrosine kinase signaling inhibitor, is essential for maintenance of the quiescent satellite cell pool (Shea et al., 2010). Spry1 is expressed in quiescent satellite cells in uninjured muscle that results in inhibition of FGF signaling to protect satellite cells from activation. Spry1 expression decreased in proliferating myoblasts after injury. Intriguingly, this expression was restored in self-renewed quiescent satellite cells. In addition, the absence of the *Spry1* gene has shown a reduction in satellite cell number after muscle regeneration, suggesting that Spoutry-1 is required for the return of myogenic precursor cells to a quiescent state through inhibition of FGF signaling and replenishment of the satellite cell pool during muscle regeneration (Shea et al., 2010). Six1, which belongs to the Six homeoprotein family, is a principal regulator of embryonic myogenesis as an upstream regulator of myogenic factors including Pax3, Myf5, and Myogenin (Giordani et al., 2007; Grifone et al., 2007). Six1 was shown as a regulator of myogenic differentiation as well as satellite cell self-renewal through Dusp6 and ERK1/2 signaling (Le Grand et al., 2009). Six1 is expressed in both quiescent and activated satellite cells (Le Grand et al., 2009). Cell transplantation experiments demonstrated that Six1 regulates myogenic differentiation through up-regulation of MyoD and Myogenin expression. Importantly, the absence of Six1 in satellite cells interfered with muscle regeneration, but led to an increase in the number of satellite cells, indicating that Six1 is required to replenish the satellite cell pool during muscle regeneration. Interestingly, Six1 inhibits extracellular signal-regulated kinase 1/2 (ERK1/2) signaling by up-regulation of Dusp6, an inhibitor of ERK1/2 signaling, which is important for the satellite cell activation process (Le Grand et al., 2009). A previous study presented that angiopoietin-1 (Ang1) and its receptor Tie-2, mediated by ERK1/2 signaling, promotes satellite cell self-renewal (Abou-Khalil et al., 2009), suggesting that the activation of ERK1/2 signaling is essential for satellite cell self-renewal, and that Six1-mediated ERK1/2 signaling inhibition is essential for satellite cell activation. Taken together, the factors that down-regulate MyoD and up-regulate Pax7 could be crucial to guiding satellite cells from an active to a quiescent state. However, it still remains to be elucidated what factors selectively induce asymmetric or symmetric division of myogenic precursor cells.

## Satellite cell asymmetric division

Kuang and colleagues have revealed the heterogeneity and the “stem cell” of satellite cells using *Myf5-Cre:Rosa26-YFP* mice, which can trace Myf5 expression (Kuang et al., 2007). After single muscle fiber isolation from *Myf5-Cre:Rosa26-YFP* mice, approximately 10% of Pax7(+) quiescent satellite cells were YFP(−) cells, indicating that these YFP(−) quiescent satellite cells have never expressed Myf5. Since Myf5 is one of the myogenic regulatory factors and known to regulate embryonic myogenesis, YFP(−) satellite cells may be less committed cells compared to the YFP(+) population. In addition, the transplantation experiments clearly revealed that Pax7(+)YFP(+) cells preferentially underwent myogenic differentiation while Pax7(+)YFP(−) cells were able to extensively contribute to generate satellite “stem cell” in regenerating muscle (Kuang et al., 2007). Interestingly, Pax7(+)YFP(−) cells were able to asymmetrically divide into both Pax7(+)YFP(−) satellite “stem cells” and Pax7(+)YFP(+) muscle committed progenitor cells (Kuang et al., 2007), while Pax7(+)YFP(+) cells never generated Pax7(+)YFP(−) satellite “stem cells.” Based on this study, Pax7(+)YFP(−) satellite “stem” cells have never expressed the *Cre* gene inserted in the *Myf5* locus, whereas recent studies have revealed that Myf5 or MyoD-expressing myogenic cells also have the potential to develop self-renewable satellite stem cells (Kuang et al., 2007). Inactivation of *Pax7* in *Myf-5*(+) lineage using *Myf5^Cre^*/^+^/*Pax7^loxP/loxP^* mice revealed that the majority of adult satellite cells originate from Myf5-expressing myogenic cells, and that the majority of satellite stem cells are replenished from Myf5-expressing cells (Gunther et al., 2013). In addition, *Myf5^Cre^*/^+^/*Pax7^loxP/loxP^*/MyoD^−/−^ mice revealed that MyoD(+)/Myf5(−) myogenic progenitor cells also give rise to Pax7-positive satellite cells during development (Gunther et al., 2013). Furthermore, Kanisicak and colleagues have shown that, using *MyoD^iCre^*/^+^/*R26R-EYFP* lineage tracing system, almost all satellite cells in adult muscle had activated MyoD once during satellite cell development, while MyoD expression is shut down in adult satellite cells (Kanisicak et al., 2009). These results strongly indicate that Myf5 and/or MyoD-expressing myogenic cells are the origin of satellite cells and satellite stem cells. Thus, it is still an open question as to how expression of Myf5 and MyoD are down-regulated in satellite stem cells during satellite cell development and self-renewal.

The idea that a portion of satellite cells are asymmetrically dividing during mitosis has been proven by multiple studies. Numb, an antagonist of the Notch signaling pathway, is asymmetrically localized during satellite cell mitosis; one daughter cell inherited a high level of Numb, and the other daughter cells had little or no Numb. Selectively Numb-expressed cells are capable of self-renewal (Conboy and Rando, 2002; Shinin et al., 2006). In addition, Shinin and colleagues have proposed by pulse chase labeling cells that template “immortal” chromosomes cosegregate into the stem-like daughter cells, while newly synthesized chromosomes are inherited by the differentiated daughter cells during asymmetric divisions of satellite cells (Shinin et al., 2006). This is based on the idea proposed by John Cairn that the oldest DNA strands of each chromosome in some stem cell populations were effectively immortal during mitosis to prevent genome mutation from continuous DNA replication (Cairns, 1975), suggesting that stem cells perform consecutive asymmetric divisions. A recent study has demonstrated that, using Pax7-nGFP mice, Pax7-nGFP^High^ muscle stem cells have a low metabolic rate and that only proliferating Pax7-nGFP^High^ cells asymmetrically segregate old DNA strands to the renewing stem cells or committed cells, indicating that Pax7-nGFP^High^ cells possess a high capacity to self-renew by inheriting old DNA strands (Rocheteau et al., 2012).

In summary, as described here, the accumulating evidence has revealed that satellite cells are a heterogeneous population, while it is still not clear how to separately isolate functional satellite “stem cell” populations and muscle committed satellite cells without using a transgene, and it remains totally unknown whether these “satellite stem cells” are homogeneous or still heterogenous populations. Further studies will be required to progress toward more specific means of identifying and isolating “stem cell” populations within satellite cells such as through the use of FACS with endogenous cell surface markers. Moreover, satellite cells asymmetrically or symmetrically are divided to generate myogenic committed progenitor cells as well as to replenish satellite stem cell pools that contribute to extensive muscle growth and regeneration. Currently, many groups attempt to elucidate the molecular and biological mechanisms of satellite cell self-renewal, and what factors selectively induce asymmetric or symmetric division of satellite cells.

## Niches and satellite cell self-renewal: satellite cell-to-matrix interactions

The concept of a stem cell niche entails the idea that cell behavior and fate is determined by the microenvironment in which stem cells exist. For example, special niches may maintain stem cells in a quiescent state while, upon injury, the microenvironment surrounding the quiescent stem cells changes, and activates stem cells to induce cell proliferation and differentiation or to promote self-renewal (Ohlstein et al., 2004; Scadden, 2006). Accumulating evidence demonstrates that multiple factors within these specific niches, which include cell-to-cell or cell-to-matrix interactions, autocrine/paracrine signaling as well as biochemical environment, are required to regulate stem cell specification (Ohlstein et al., 2004; Scadden, 2006). Recently several studies have emerged to define the satellite cell niche as well as the underlying mechanisms of how the niche regulates satellite cell self-renewal (Table 1).

**Table 1:** **Regulatory molecules and niches for satellite cell self-renewal**.

**Factor**	**Localization**	**Downstream target**	**Function on satellite cell**	**References**
Par complex	ASC	Activate p38 α/β MAPK	Par(+): myogenic differentiation Par(−): self-renewal	Troy et al., 2012
miR-489	QSC	Suppress Dek expression	Maintain satellite cell in quiescent state	Cheung et al., 2012
Dek	ASC	Chromatin remodeling	Promote satellite cell activation	Cheung et al., 2012
Sprouty-1 (Spry1)	QSC	Tyrosine kinase inhibitor of FGF signaling	Maintain satellite cell number	Shea et al., 2010
FGF2	Aged myofiber	Promote cell proliferation	Niche to maintain satellite cell number	Chakkalakal et al., 2012
Six1	QSC, ASC	Negatively regulate ERK1/2 signaling	Induce satellite cell self-renewal and differentiation	Le Grand et al., 2009
Angiopoietin 1 (Ang1)/Tie2	QSC	Activate ERK1/2 signaling	Autocrine and paracrine effects to maintain satellite cell number	Abou-Khalil et al., 2009
Wnt7a/Fzd7	QSC	Wnt7a/PCP pathway	Symmetric division of satellite stem cell	Le Grand et al., 2009
Fibronectin	ASC	Bind with Syndecan-4/Fzd7 to induce Wnt7a-signaling	Symmetric division of satellite stem cell	Bentzinger et al., 2013
Syndecan-4	QSC	Enhance Wnt7a-signaling	Symmetric division of satellite stem cell	Bentzinger et al., 2013
Notch3	QSC	Notch signaling	Maintain satellite cell number	Fukada et al., 2007; Kitamoto and Hanaoka, 2010
Hesr1/Hesr3	QSC	Downstream of Notch	Maintain satellite cell number	Fukada et al., 2011
Numb	ASC	Downstream of Notch	Numb(+): differentiation Numb(−): self-renew	Conboy and Rando, 2002
RBP-J	QSC, ASC	Co-activator of notch signaling	Maintain satellite cell number	Bjornson et al., 2012; Mourikis et al., 2012
MEGF10	QSC, ASC	Activate Notch signaling	Maintain satellite cell number	Holterman et al., 2007
Collagen VI	ECM for QSC	Increased muscle stiffness	Maintain satellite cell self-renewal	Urciuolo et al., 2013
Hypoxia	Outside of QSC	Down-regulate miR-1/206 Surpresse MyoD expression	Maintain satellite cell in quiescent state	Liu et al., 2012

QSC, quiescent satellite cells; ASC, activated satellite cells; RC, reserve cells; ECM, extracelllular matrix.

Wnt signaling pathways play key roles in the regulation of multiple developmental processes, from embryonic myotome induction, to postnatal myogenic differentiation (Rochat et al., 2004; Gros et al., 2009). In addition, Wnt signaling acts as a protagonist for myogenic specification in muscle resident stem cells (Polesskaya et al., 2003; Brack et al., 2007). Activation of Wnt signaling is normally initiated through binding with the G protein-coupled receptor proteins of the Frizzled (Fzd) family. Activated Wnt signaling branches into two distinct pathways, the canonical Wnt/β-catenin pathway, and the non-canonical Wnt/planar cell polarity (PCP) pathway. The canonical Wnt pathway induces satellite cell proliferation during muscle regeneration (Otto et al., 2008), whereas the non-canonical Wnt pathway induces myocyte growth in the developing myotome (Gros et al., 2009). Recent studies have presented that one of the Wnt receptors, Fzd7, is highly expressed in Pax7(+)Myf5(−) quiescent satellite stem cells, and that Wnt7a is a ligand for Fzd7. Intriguingly, Wnt7a/Fzd7 promoted the symmetric division of a Pax7(+)Myf5(−) satellite stem cell population through the activation of the non-canonical Wnt pathway (Le Grand et al., 2009). Additionally, overexpression of Wnt7 enhanced muscle regeneration with an expansion of satellite stem cell number, while overexpression of Wnt3, which stimulated the canonical Wnt pathway, impaired muscle regeneration. In addition, recent studies have found that Syndecan-4 and Fzd7 form a co-receptor complex for Wnt7a in activated satellite cells, and that binding of the ECM glycoprotein, Fibronectin, to Syndecan-4 promoted Wnt signaling to induce the symmetric expansion of satellite stem cells (Bentzinger et al., 2013). These observations suggest that the activation of the non-canonical Wnt pathway through fibronectin controls the symmetric division of satellite cells and replenishes the satellite cell pool during muscle regeneration.

The Notch signaling pathway is also known to be important for various cell functions, including cell-cell interaction and cell fate determination during tissue development, homeostasis, and regeneration (Lai, 2004). Activation of Notch signaling prevents myoblast differentiation and promote satellite cell self-renewal by repressing MyoD activity through up-regulation of HES proteins, an inhibitor of MyoD (Kuroda et al., 1999). By contrast, attenuation of Notch signaling by Numb, a Notch inhibitor, results in the progression of myogenesis from myoblast proliferation to differentiation (Conboy and Rando, 2002). Additionally, a recent paper has demonstrated that absence of *Numb* in *Pax7-CreER/Numb^fl/fl^* mice results in a satellite cell proliferation defect due to up-regulation of cell cycle inhibitor p21 and Myostatin and display the severe impairment of muscle regeneration, indicating a unique role for Numb in regulating the activation and proliferation of satellite cells (George et al., 2013). In fact, Numb expression was asymmetrically localized in satellite stem cells during satellite cell activation. Thus, the balance between Notch1 and Numb regulates satellite cell proliferation, differentiation, and self-renewal (Conboy and Rando, 2002). In regenerating aged muscle, the disruption of Notch activity extensively impairs muscle regeneration, whereas activation of Notch signaling promotes muscle regeneration (Conboy et al., 2003). Microarray analysis identified that Notch3 is highly expressed in quiescent satellite cells (Fukada et al., 2007), and *Notch3*-deficient mice presented an increased number of Pax7(+) self-renewing cells (Kitamoto and Hanaoka, 2010). In addition, the absence of Notch signaling in satellite cells of adult mice induces spontaneous activation from the quiescent state to rapid differentiation, resulting in depletion of the satellite cell pool and the failure of muscle regeneration (Bjornson et al., 2012; Mourikis et al., 2012). Moreover, Hesr1 and Hesr3, which are downstream target genes of Notch signaling, are expressed in quiescent satellite cells. The absence of both Hesr1 and Hesr3 genes also leads to the reduction of satellite cell self-renewal and the depletion of the satellite cell pool, leading to the consequent impairment of muscle regeneration (Fukada et al., 2011). A recent paper demonstrated that mice lacking the *RBP-J* gene, an effector transcription factor of Notch signaling, display the depletion of the satellite cell pool, and this satellite cell depletion is recovered in *MyoD*^−/−^ background (*coRbpj;MyoD*^−/−^) (Brohl et al., 2012). However, in *coRbpj;MyoD*^−/−^ mice, satellite cells are not located in the normal position, and are instead positioned in the interstitial space of muscle fibers, and do not contribute to myofiber growth (Brohl et al., 2012). Furthermore, microarray analysis revealed that Notch signaling is required to produce sufficient numbers of basement membrane proteins and adhesion molecules including α7-Integrin, Collagen XVIIIa1, Megf10, and M-CAM (Table 1). One of them, MEGF10 was found to be expressed in quiescent and activated satellite cells, and overexpression of MEGF10 stimulates the generation of quiescent satellite cells (Holterman et al., 2007). These observations strongly suggest that Notch signaling contributes to maintenance of the satellite cell pool by repressing MyoD as well as by promoting the homing of satellite cells along with stimulation of basal lamina production.

In normal skeletal muscle, the distributions of satellite cells are anatomically observed close to capillaries, and the paracrine signaling of endothelial cells stimulates muscle satellite cell growth (Christov et al., 2007), suggesting that the maintenance and proliferation of satellite cells may be influenced by the interaction between endothelial cells and satellite cells. A recent study revealed that Ang1/Tie2 signaling, which are the regulatory factors of vascular homeostasis, are involved in the regulation of satellite cell self-renewal (Abou-Khalil et al., 2009). Ang1 and Tie2 expression is high in quiescent satellite cells as well as in reserve cells. In addition, inhibition of Tie2 attenuated the number of quiescent satellite cells, whereas activation of Ang1/Tie2 signaling through the ERK1/2 pathway increased the generation of quiescent satellite cells. In addition, secreted Ang1 from vascular smooth muscle cells or fibroblasts located around satellite cells promotes Pax7 expression (Abou-Khalil et al., 2009). These observations suggest that both autocrine and paracrine Ang-1 mediated Tie2 signaling is required to regulate satellite cell self-renewal. Although it is still not clear whether the direct connection between endothelial cells and satellite cells is critical for niches to maintain the satellite cell pool, it is an attractive hypothesis that stem cell niches are generated by endothelial cells surrounding satellite cells, similar to the other stem cell systems including hematopoietic stem cells and neural stem cells (Gomez-Gaviro et al., 2012).

Since satellite cells are normally located in contact with the plasma membrane and the basal lamina of muscle fibers, the association of satellite cells with muscle fibers is also considered to be an important niche for maintaining the satellite cell pool (Bischoff, 1990). In ageing muscle, the number of satellite cells and the muscle regeneration ability were decreased (Shefer et al., 2006; Collins et al., 2007) due to the changing niches in aging muscles fibers (Conboy et al., 2003; Brack et al., 2007; Carlson et al., 2008), suggesting that the stem cell niche is responsible for the maintenance of quiescent satellite cells. Therefore, the essential factors to maintaining quiescent satellite cells and thus muscle homeostasis may exist in muscle fibers. Recent studies have revealed that increased levels of FGF2, a member of the fibroblasts growth factor family, induced the loss of satellite cell number in aged muscle fibers (Chakkalakal et al., 2012). The FGF family is a well-characterized growth factor, and considered to be a stimulator of satellite cell activation and proliferation (Sheehan and Allen, 1999). This increase in FGF signaling prevents the withdrawal of satellite cells from the cell cycle, resulting in reduced satellite cell numbers. Furthermore, as described above, continuous FGF signaling through the absence of Spry-1, an inhibitor of FGF signaling, has induced the loss of satellite cells, suggesting that FGF signaling through the stem cell niche is crucial to the maintenance of the satellite cell pool during aging (Sheehan and Allen, 1999).

The extracellular matrix (ECM) surrounding muscle cells is also known to undergo extensive remodeling during muscle regeneration. Consequently, excessive composition of interstitial ECM promotes the appearance of connective tissue or fibrosis in muscle under certain conditions such as those seen in DMD or aging muscles. Urciuolo and colleagues have recently demonstrated that an ECM protein, collagen VI, is critical as an extracellular niche protein to maintain the satellite cell pool (Urciuolo et al., 2013). Mice having an absence of collagen VI (*Col6a1*^−/−^) showed delayed muscle regeneration and reduced satellite cell self-renewal (Urciuolo et al., 2013). Importantly, transplantation of fibroblasts, isolated from wild-type mice into *Col6a1*^−/−^ mice, could rescue muscle satellite cell self-renewal; this indicates that a muscle environment consisting of ECM components, including Collagen VI, can modulate satellite cell behavior and that ECMs are critical factors in maintaining the muscle satellite cell pool.

As described above, accumulating evidence has elucidated the signaling network for self-renewal in satellite cells while Gilbert and colleagues provide us new insight that the skeletal muscle microenvironment potentially regulates stem cell fate (Gilbert et al., 2010). They have engineered a hydrogel platform, which can recapitulate muscle physiological elasticity *in vitro*. Satellite cells cultured on this substrate have shown higher potency of self-renewal than those cultured on traditional plastic dishes. Additionally, myoblasts cultured on hydrogel could promote the efficacy of cell engraftment and the reconstitution of satellite stem cells *in vivo*, indicating that substrate elasticity is also an important factor of satellite cell self-renewal (Gilbert et al., 2010).

In addition to cell commitment, recent reports indicate that physiological conditions in satellite cells may influence the regulation of self-renewal. Liu and colleagues have proposed that hypoxia does not affect myoblast proliferation but instead promotes satellite cell self-renewal through up-regulating Pax7 (Liu et al., 2012). In fact, hypoxia promotes the activation of Notch signaling, leading to the down-regulation of miRNA-1/206 expression. Since miRNA-1/206 can bind to its target site on the Pax7-3'UTR, decreased levels of both miRNAs through hypoxic conditions can induce Pax7 expression (Liu et al., 2012). In addition, a previous study demonstrated that hypoxic conditions strongly reduce MyoD and myogenin expression during myoblast differentiation without inducing apoptotic cell death (Majmundar et al., 2012), suggesting that the hypoxic condition mediating Notch signaling and microRNAs could be an important factor in regulation of MyoD and Pax7 for the maintenance of the satellite cell pool.

## Conclusions

It is widely accepted that satellite cells are essential for postnatal muscle growth and muscle regeneration. Despite high potency of myogenic differentiation in satellite cells, these cells are currently not applicable for cell transplantation therapy against DMD due to severe limitations, such as low cellular survival, incomplete myogenic differentiation, and especially poor satellite cell formation (Tedesco and Cossu, 2012). Alternatively, mesenchymal stem cells including bone marrow derived stem cells were expected to be a new cell source for cell therapy. However, since the transplantation efficacy is quite low, stem cell therapy in muscular dystrophy is still an elusive goal. To accomplish effective cell therapy, injected cells must build and maintain the satellite cell pool with continuous self-renewal. In order to solve these issues, it is essential to understand the mechanisms of muscle regeneration through satellite cells and non-satellite cells.

Recently, accumulating evidence has revealed the satellite cell heterogeneity and the molecular mechanisms of cell fate determination, specifically whether satellite cells differentiate into myocytes or self-renew as stem cells. These studies demonstrated that maintenance of the satellite cell pool is intrinsically and extrinsically regulated by many regulators. Novel knowledge about muscle regeneration through satellite cells may provide us new therapeutic approaches for DMD patients. Additionally, there are multiple unexplained muscular diseases, such as sarcopenia or muscle atrophy. In order to discover ideal therapies for muscular diseases, it is essential to explore fundamental molecular mechanisms of muscle satellite cells using new methodological technologies such as sequencing-mediated global gene regulation.

## Author contributions

Norio Motohashi and Atsushi Asakura wrote the manuscript. Both authors read and approved the final manuscript.

### Conflict of interest statement

The authors declare that the research was conducted in the absence of any commercial or financial relationships that could be construed as a potential conflict of interest.

## Abbreviations

DMD: Duchenne muscular dystrophy
bHLH: Basic-helix-loop-helix
mRNP: Messenger ribonucleoprotein
FACS: Fluorescence-activated cell sorting
UTR: Untranslated region
MAPK: Mitogen-activated Protein Kinase
PKC: Protein kinase C
Spry1: Sprouty homolog 1
FGF: Fibroblast growth factors
Dusp6: Dual specificity phosphatase 6
ERK: Extracellular signal-regulated kinase
Ang1: Angiopoietin-1
Fzd7: Frizzled 7
PCP pathway: planar cell polarity pathway
ECM: *Extracellular* matrix
M-CAM: Melanoma cell adhesion molecule.
